# 903. Closing the Gap: A Practical Solution to Clothing Barriers in Burn Care Transitions

**DOI:** 10.1093/jbcr/irag033.399

**Published:** 2026-04-06

**Authors:** Leah Rahman, Stacey Dawson

**Affiliations:** Warden Burn Center at Orlando Health Orlando Regional Medical Center, Ocoee, FL; Warden Burn Center at Orlando Health Orlando Regional Medical Center, Lakeland, FL

## Abstract

**Introduction:**

Burn patients frequently encounter significant social challenges upon discharge, particularly those affected by house fires, motor vehicle accidents, or homelessness. One critical barrier is the lack of clothing. Many patients experience significant socioeconomic hardships, including homelessness, displacement due to residential damage or lack of insurance.

By acknowledging the often-overlooked issue of inadequate clothing, the purpose of this project emphasized a holistic model of burn care, one that goes beyond medical treatment to include essential social and emotional support. In response, our burn unit initiated a staff-led clothing drive to provide essential garments for patients in need. This initiative aims to ensure access to clean, seasonally appropriate clothing, supporting discharges.

**Methods:**

A clothing drive was initiated by the Clinical Assistant Nurse Manage in May 2024. The drive was promoted through weekly emails and in-person announcements via huddles and team summits. To encourage contributions, burn closet donation inventory was strategically requested around holidays and spring-cleaning periods. A structured process was implemented to collect, organize, store, and distribute clothing from staff and their families. Patients in need of clothing were identified through routine nursing rounds and direct nurse referrals. When patients voiced concerns about lacking appropriate attire to the burn team, nurses promptly flagged these needs to ensure timely support. This approach helped address clothing needs in a responsive and compassionate manner.

**Results:**

From May 2024 through May 2025, the clothing closet initiative supported about 165 patients out of 220 individuals served. This resource provided essential clothing items to adult patients ranging in age from 24 to 67, encompassing both male and female recipients. Staff participation was high, and donation volumes increased during targeted promotion periods. Patients expressed gratitude, and discharge planning was facilitated by the availability of clothing.

**Conclusions:**

Providing clothing at discharge is a simple yet impactful intervention that supports patient dignity and readiness for transition from the hospital. This initiative demonstrates how unit-level efforts can address social determinants of health and best prepare vulnerable populations for discharge.

**Applicability of Research to Practice:**

This model can be replicated in other burn units or healthcare settings to support vulnerable populations. It highlights the importance of addressing non-clinical barriers in discharge planning and encourages staff engagement in holistic patient care.

**Funding for the study:**

N/A.

